# Supplementary material for: Neutrality in the Metaorganism
Source: PLoS Biol. 2019 Jun 19;17(6):e3000298. doi: 10.1371/journal.pbio.3000298 (PMC6583948; doi:10.1371/journal.pbio.3000298)
Supplement: S1 Table — Best-fit data are the mean values from 100 rarefactions at a level of 1,000 reads per sample. Reference is to the original study containing detailed information on sample preparation and raw sequencing data. The data summarized in this table are also available in S1 Data. (PDF) [file pbio.3000298.s001.pdf]

Samples	Source	#samples	#OTUs	best-fit m	R^2 neutral	AIC neutral	AIC binomial	Reference
Caenorhabditis elegans								[1]
natural samples	natural	22	193	0.03	0.37	-673.2	-452.5	
lab samples	lab	34	106	0.01	0.33	-330.0	-208.2	
Ircinia oros								
Spain, Barcelona	natural	11	1121	0.41	0.6	-5391.47	-4966.26	[2]
Sarcotragus fasciculatus								
Spain, Barcelona	natural	12	735	0.83	0.81	-3552.82	-3165.09	[2]
Carteriospongia foliascens								[2]
Australia, Davies Reef	natural	15	939	0.36	0.6	-5435.40	-5007.39	
Australia, Fantome Island	natural	14	728	0.81	0.79	-3740.45	-3336.08	
Australia, Orpheus Island	natural	15	750	0.86	0.8	-3987.01	-3539.84	
Australia, Green Island	natural	13	774	0.47	0.59	-3846.0	-3542.79	
Australia, Torres Strait	natural	7	336	0.78	0.58	-1410.75	-1244.0	
Mus musculus								[3]
natural samples	natural	69	281	0.11	0.84	-1217.97	-998.15	
lab samples	lab	54	136	0.18	0.82	-536.30	-452.48	
Nematostella vectensis								[4]
1 day post fertilization (dpf)	lab	6	226	0.23	0.46	-738.68	-655.06	
4 dpf	lab	15	149	0.33	0.67	-521.62	-479.29	
40 dpf	lab	12	155	0.09	0.59	-502.39	-413.96	
123 dpf	lab	12	195	0.39	0.69	-678.72	-623.49	
385 dpf	lab	20	225	0.17	0.58	-766.93	-679.74	
401 dpf	lab	8	120	0.7	0.74	-446.34	-402.59	
Hydra vulgaris								[5]
0.5 weeks after hatching (wah)	lab	8	699	0.6	0.54	-2527.91	-2289.35	
2.5 wah	lab	8	248	0.28	0.44	-820.92	-756.19	
5 wah	lab	8	242	0.53	0.45	-1129.85	-1041.28	
9 wah	lab	8	257	0.5	0.43	-833.46	-765.88	
15 wah	lab	8	140	0.45	0.59	-466.20	-432.55	
Aurelia aurita								[6]
control	lab	5	163	0.91	0.69	-624.96	-538.45	
quorum quenching (QQ)	lab	18	391	0.62	0.85	-1716.09	-1532.6	
Environment								
Compost	natural	65	587	0.48	0.87	-2690.26	-2491.77	[1]
Seawater	natural	16	2518	0.62	0.7	-12345.33	-11113.17	[2]
Sediment	natural	12	3796	0.77	0.47	-16658.27	-14665.48	[2]

References

[1] Dirksen P, Marsh SA, Braker I, Heitland N, Wagner S, Nakad R, et al. The native microbiome of the nematode *Caenorhabditis elegans*: gateway to a new host-microbiome model. BMC Biology. 2016;14(1):38.

[2] Thomas T, Moitinho-Silva L, Lurgi M, Björk JR, Easson C, Astudillo-García C, et al. Diversity, structure and convergent evolution of the global sponge microbiome. Nature Communications. 2016;7:11870.

[3] Wang J, Kalyan S, Steck N, Turner LM, Harr B, Künzel S, et al. Analysis of intestinal microbiota in hybrid house mice reveals evolutionary divergence in a vertebrate hologenome. Nature Communications. 2015;6:6440.

[4] Mortzfeld BM, Urbanski S, Reitzel AM, Künzel S, Technau U, Fraune S. Response of bacterial colonization in *Nematostella vectensis* to development, environment and biogeography. Environmental Microbiology. 2016;18(6):1764–1781.

[5] Franzenburg S, Fraune S, Altrock PM, Kuenzel S, Baines JF, Traulsen A, et al. Bacterial colonization of Hydra hatchlings follows a robust temporal pattern. ISME Journal. 2013;7:781–790.

[6] Weiland-Bräuer N, Fischer MA, Pinnow N, Schmitz RA. Potential role of host-derived quorum quenching in modulating bacterial colonization in the moon jellyfish *Aurelia aurita*. Scientific Reports. 2019;9(1):34.
