# Supplementary material for: Neutrality in the Metaorganism
Source: PLoS Biol. 2019 Jun 19;17(6):e3000298. doi: 10.1371/journal.pbio.3000298 (PMC6583948; doi:10.1371/journal.pbio.3000298)
Supplement: S2 Table — (PDF) [file pbio.3000298.s002.pdf]

Domain	Phylum	Class	Order	Family	Genus	
Bacteria	Bacteroidetes	Flavobacteria	Flavobacteriales	Flavobacteriaceae	unclassified	over-represented
Bacteria	Proteobacteria	Alphaproteobacteria	Rhizobiales	Hyphomicrobiaceae	Devosia	
Bacteria	Proteobacteria	Alphaproteobacteria	unclassified	unclassified	unclassified	
Bacteria	Proteobacteria	Betaproteobacteria	Burkholderiales	Alcaligenaceae	unclassified	
Bacteria	Proteobacteria	Betaproteobacteria	Burkholderiales	Oxalobacteraceae	unclassified	
Bacteria	Proteobacteria	Betaproteobacteria	Burkholderiales	unclassified	unclassified	
Bacteria	Proteobacteria	Betaproteobacteria	unclassified	unclassified	unclassified	
Bacteria	Proteobacteria	Gammaproteobacteria	Pseudomonadales	Pseudomonadaceae	unclassified	
Bacteria	Proteobacteria	Gammaproteobacteria	unclassified	unclassified	unclassified	
Bacteria	Proteobacteria	Gammaproteobacteria	Xanthomonadales	Xanthomonadaceae	unclassified	
Bacteria	unclassified	unclassified	unclassified	unclassified	unclassified	
Bacteria	Proteobacteria	Alphaproteobacteria	Rhizobiales	Brucellaceae	Ochrobactrum	under-represented
