# Supplementary material for: Neutrality in the Metaorganism
Source: PLoS Biol. 2019 Jun 19;17(6):e3000298. doi: 10.1371/journal.pbio.3000298 (PMC6583948; doi:10.1371/journal.pbio.3000298)
Supplement: S3 Table — (PDF) [file pbio.3000298.s003.pdf]

Domain	Phylum	Class	Order	Family	Genus	
Bacteria	Bacteroidetes	Bacteroidia	Bacteroidales	Bacteroidaceae	Acetomicrobium	over-represented
Bacteria	Bacteroidetes	Bacteroidia	Bacteroidales	Porphyromonadaceae	Proteiniphilum	
Bacteria	Bacteroidetes	Flavobacteria	Flavobacteriales	Cryomorphaceae	Fluviicola	
Bacteria	Firmicutes	Clostridia	Clostridiales	Clostridiaceae 4	Caminicella	
Bacteria	Firmicutes	Clostridia	Clostridiales	Clostridiales_Incertae Sedis XIII	Anaerovorax	
Bacteria	Firmicutes	Clostridia	Clostridiales	Lachnospiraceae	Clostridium XlVb	
Bacteria	Firmicutes	Clostridia	Clostridiales	Lachnospiraceae	Coprococcus	
Bacteria	Firmicutes	Clostridia	Clostridiales	Lachnospiraceae	Dorea	
Bacteria	Firmicutes	Clostridia	Clostridiales	Peptococcaceae 1	Peptococcus	
Bacteria	Firmicutes	Clostridia	Clostridiales	Ruminococcaceae	Acetanaerobacterium	
Bacteria	Firmicutes	Clostridia	Clostridiales	Ruminococcaceae	Anaerotruncus	
Bacteria	Firmicutes	Clostridia	Clostridiales	Ruminococcaceae	Ethanoligenens	
Bacteria	Firmicutes	Clostridia	Clostridiales	Ruminococcaceae	Flavonifractor	
Bacteria	Firmicutes	Clostridia	Clostridiales	Ruminococcaceae	Hydrogenoanaerobacterium	
Bacteria	Firmicutes	Clostridia	Clostridiales	Ruminococcaceae	Sporobacter	
Bacteria	Firmicutes	Negativicutes	Selenomonadales	Veillonellaceae	Anaerospora	
Bacteria	Bacteroidetes	Bacteroidia	Bacteroidales	Marinilabiaceae	Anaerophaga	under-represented
Bacteria	Bacteroidetes	Bacteroidia	Bacteroidales	Porphyromonadaceae	Butyricimonas	
Bacteria	Bacteroidetes	Bacteroidia	Bacteroidales	Porphyromonadaceae	Paludibacter	
Bacteria	Bacteroidetes	Bacteroidia	Bacteroidales	Porphyromonadaceae	Tannerella	
Bacteria	Bacteroidetes	Bacteroidia	Bacteroidales	Rikenellaceae	Rikenella	
Bacteria	Bacteroidetes	Sphingobacteria	Sphingobacteriales	Flammeovirgaceae	Sediminitomix	
Bacteria	Firmicutes	Clostridia	Clostridiales	Lachnospiraceae	Blautia	
Bacteria	Firmicutes	Clostridia	Clostridiales	Lachnospiraceae	Butyrivibrio	
Bacteria	Firmicutes	Clostridia	Clostridiales	Lachnospiraceae	Catonella	
Bacteria	Firmicutes	Clostridia	Clostridiales	Lachnospiraceae	Marvinbryantia	
