# Supplementary figures and images for: Neutrality in the Metaorganism
Source: PLoS Biol. 2019 Jun 19;17(6):e3000298. doi: 10.1371/journal.pbio.3000298 (PMC6583948; doi:10.1371/journal.pbio.3000298)

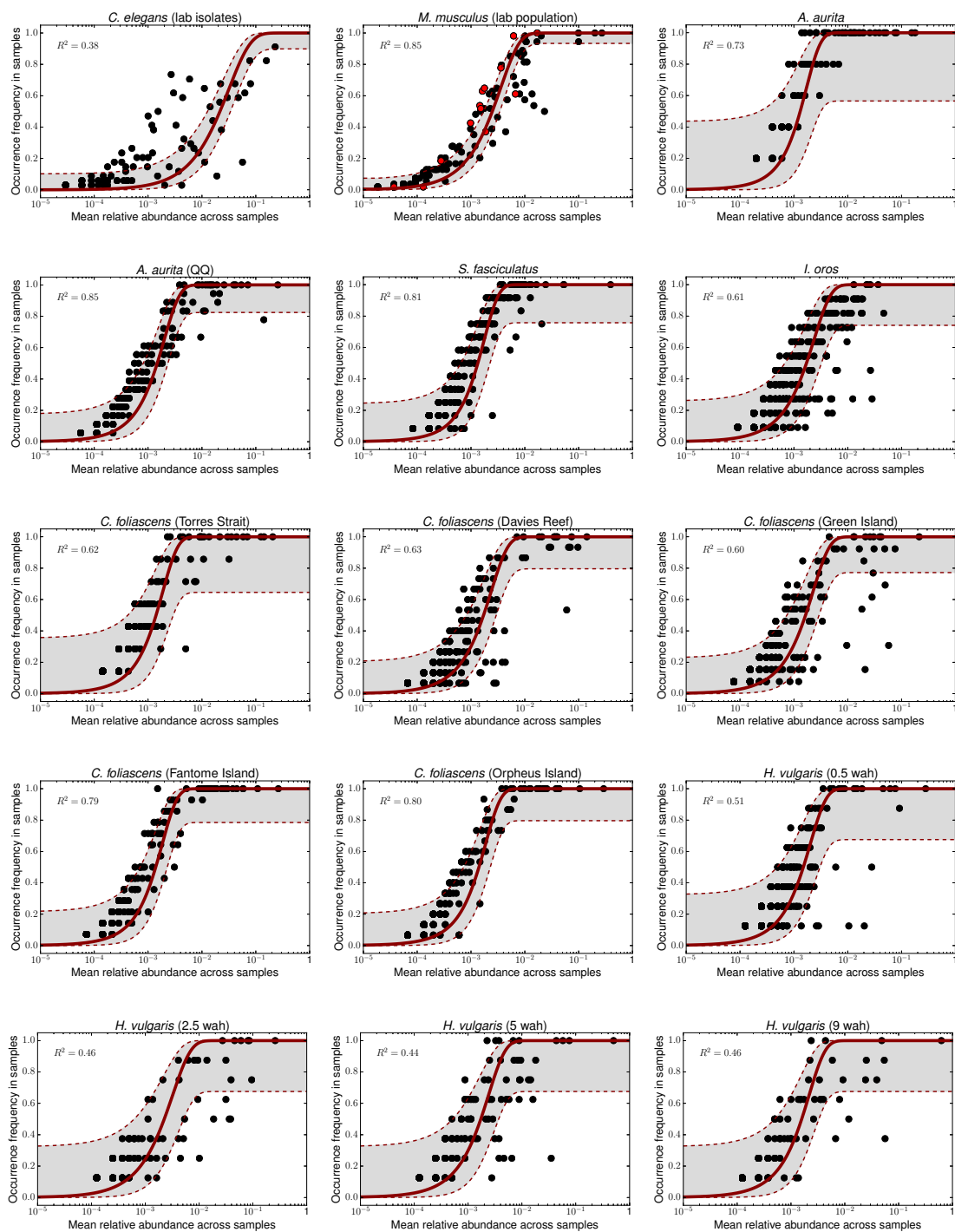

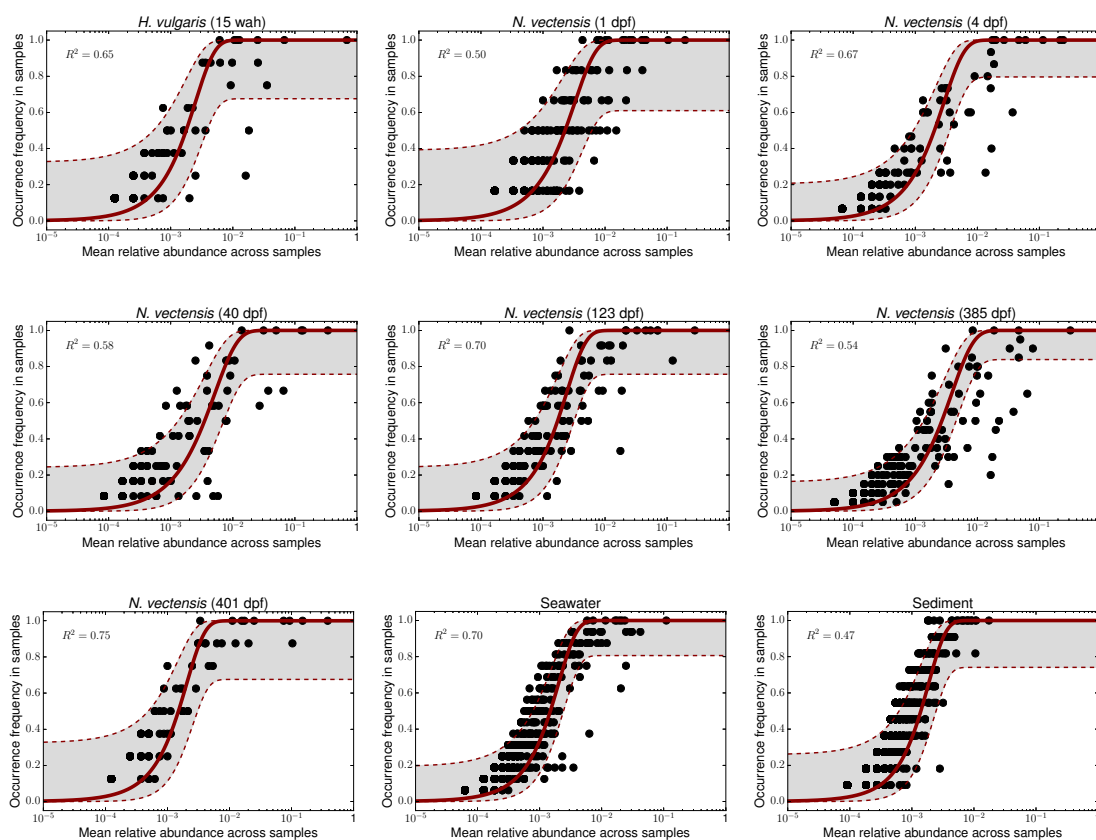

Supplement: S1 Fig — Each dot represents a taxon and the solid line is the best-fitting neutral community expectation. The dashed lines and gray area depict the 95% confidence bands. The OTU abundance data used to generate this figure are available in S2–S10 Data. OTU, operational taxonomic unit. (PDF) [file pbio.3000298.s004.pdf]

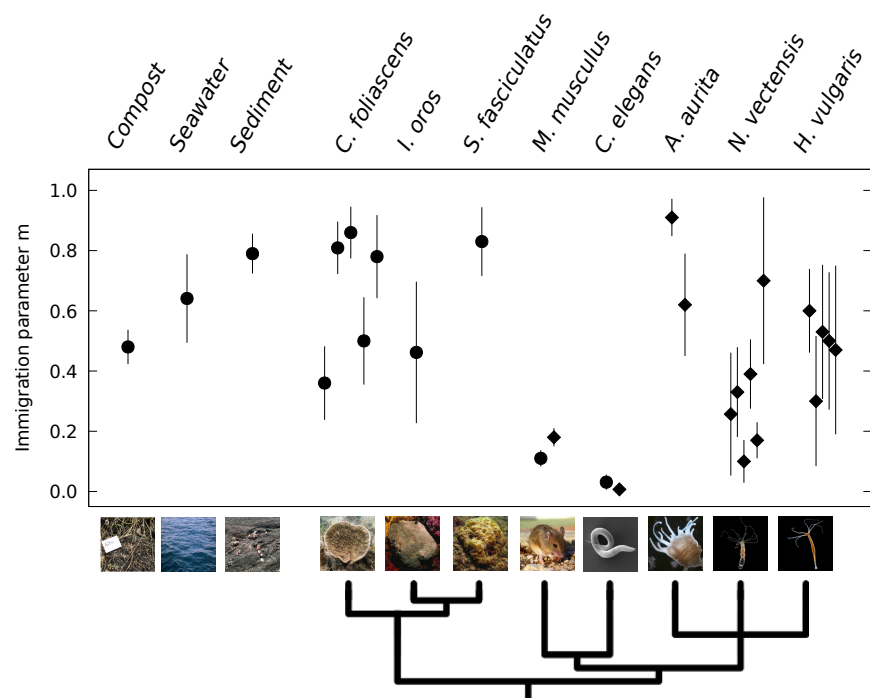

Supplement: S2 Fig — Circles denote natural populations and diamonds denote laboratory populations; error bars indicate 95% bootstrap confidence intervals. The data for C. foliascens are from several different natural populations, while the data for H. vulgaris and N. vectensis are from different time points. Spread of points along the x-axis is added to increase visibility. The data used to generate this figure are available in S1 Data. Phylogeny were generated with phyloT based on NCBI taxonomy. Sponge photographs courtesy of Susanna López-Legentil (UNC Wilmington) and Mari-Carmen Pineda (AIMS). AIMS, Australian Institute of Marine Science; NCBI, National Center for Biotechnology Information; UNC, University of North Carolina. (PDF) [file pbio.3000298.s005.pdf]

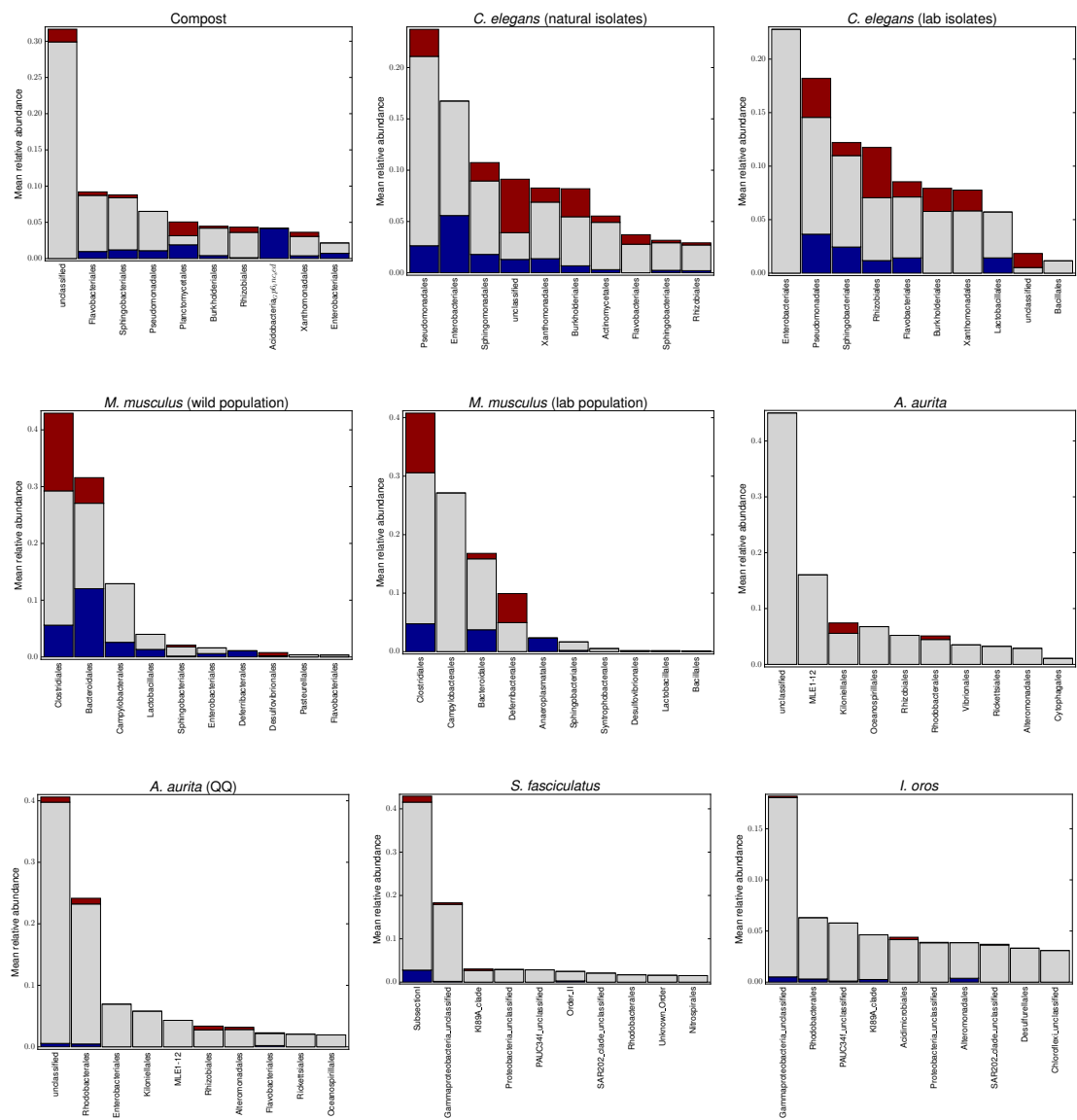

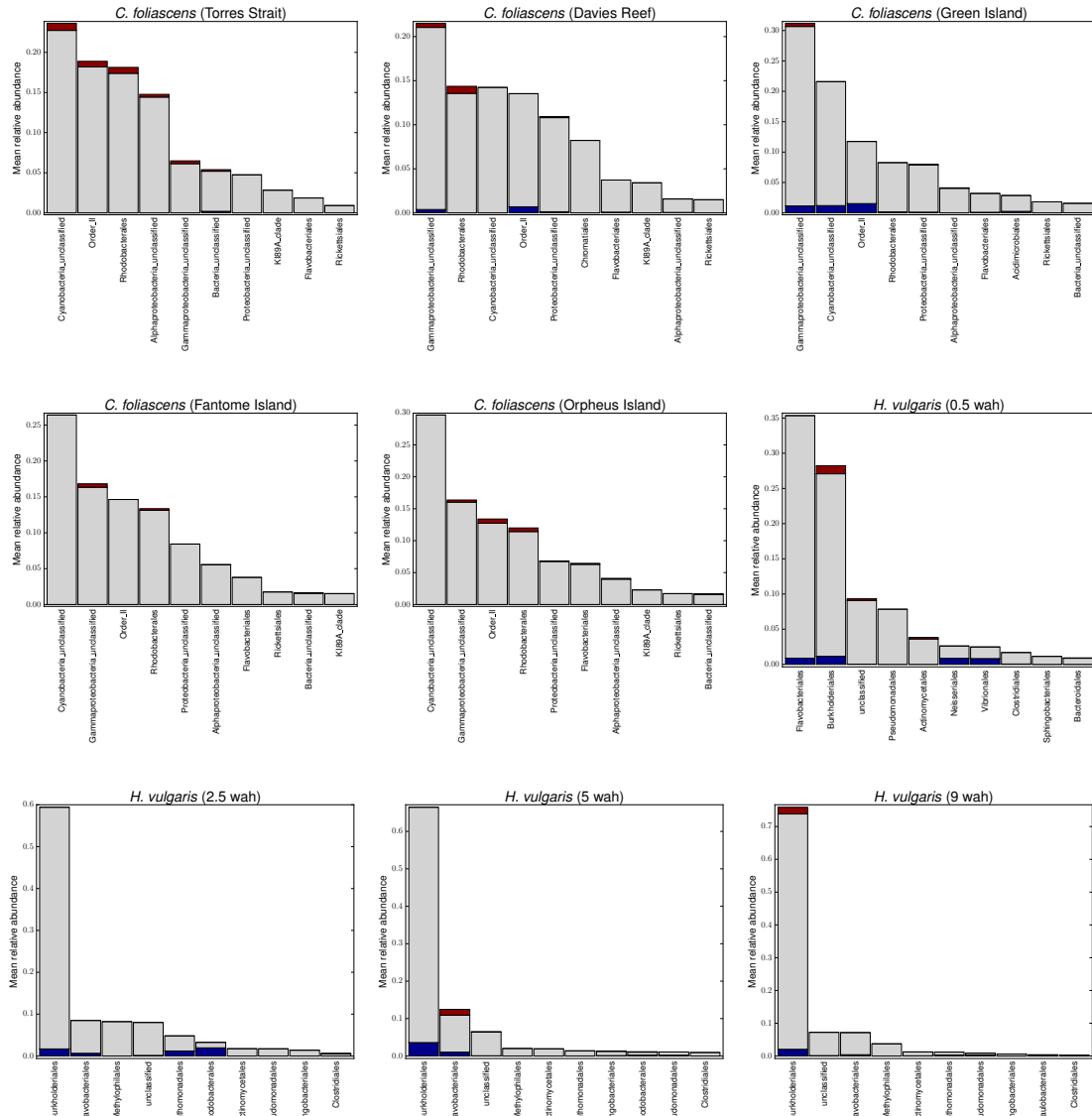

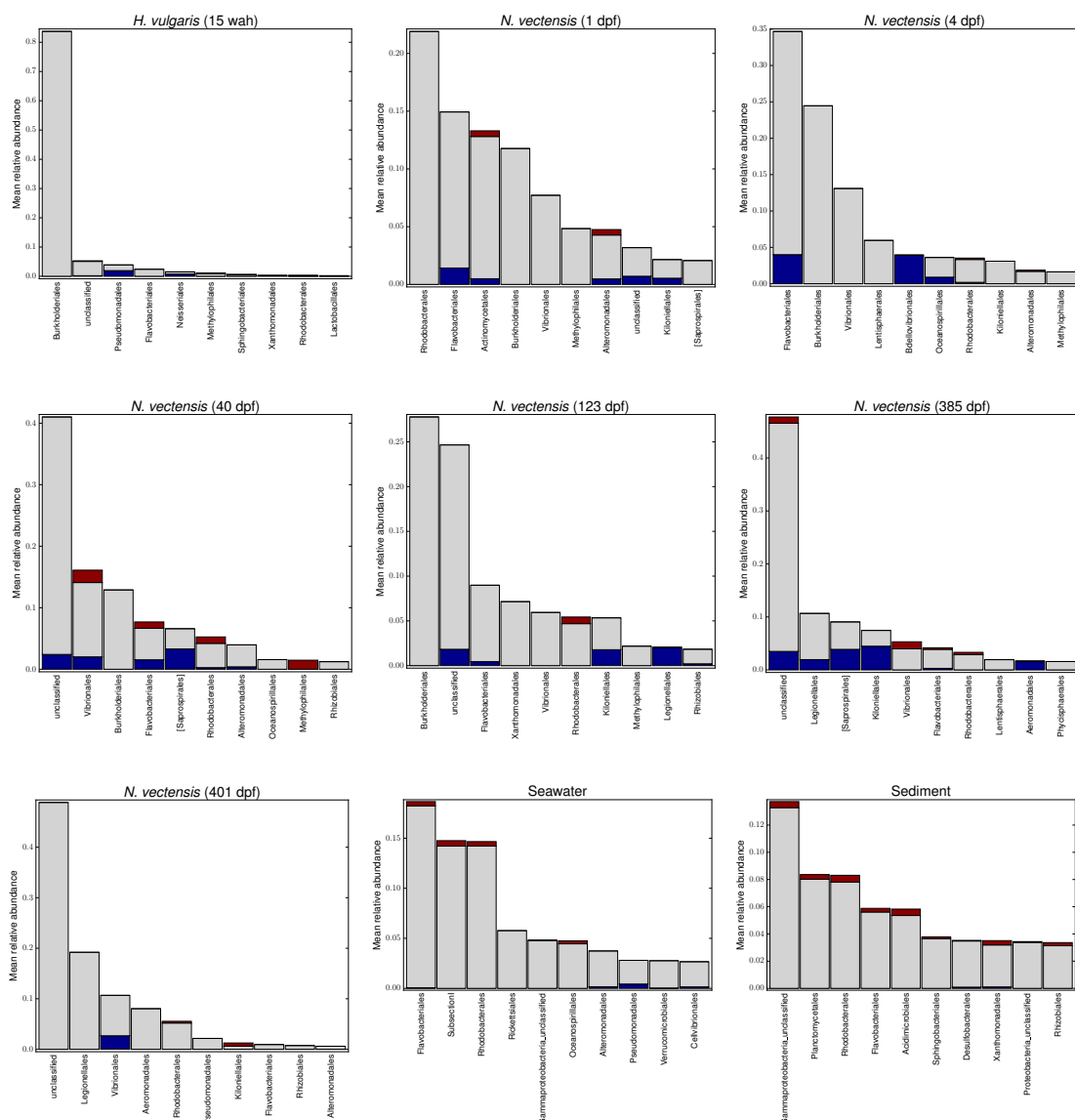

Supplement: S3 Fig — The colored sections indicate the fractions of OTUs within that order that were found above the neutral expectation (red), within the neutral expectation (gray), and below the neutral expectation (blue). The OTU abundance data used to generate this figure are available in S2–S10 Data. OTU, operational taxonomic unit. (PDF) [file pbio.3000298.s006.pdf]

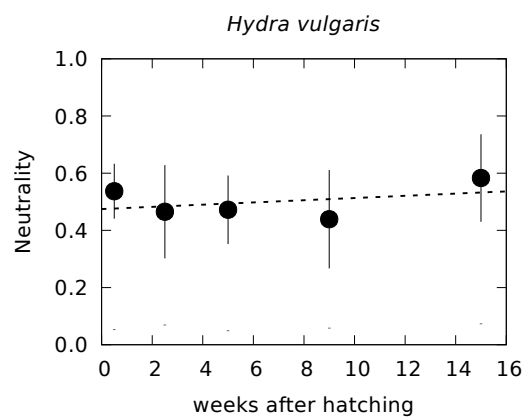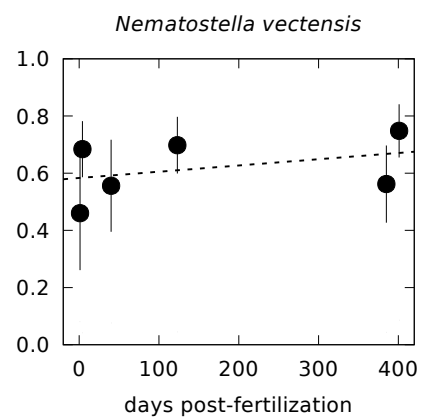

Supplement: S4 Fig — The dashed lines are the best linear fits; slopes are not significantly different from zero. The data used to generate this figure are available in S1 Data. (PDF) [file pbio.3000298.s007.pdf]

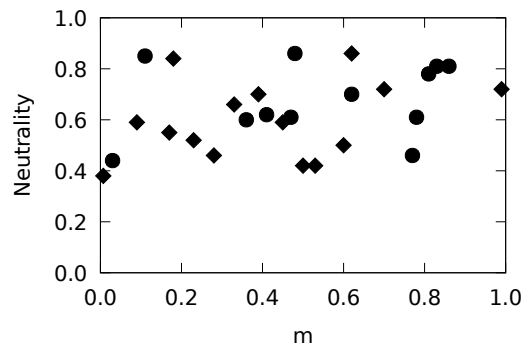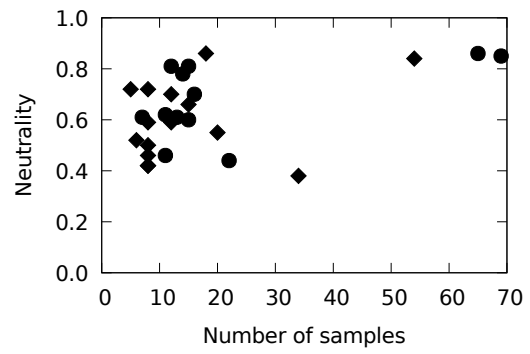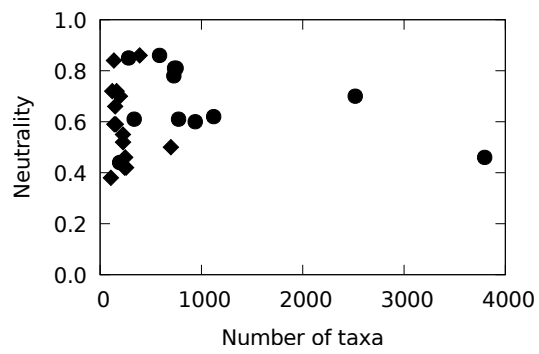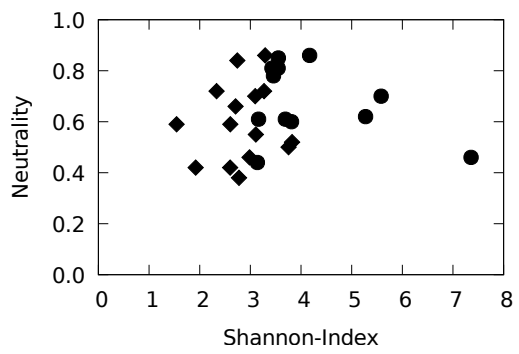

Supplement: S5 Fig — Consistency with the neutral model versus the estimated dispersal parameter m (top left), the number of samples (top right), the number of identified taxa (bottom left), and the Shannon index of diversity (bottom right). Circles denote natural populations and diamonds laboratory populations. The data used to generate this figure are available in S1 Data. (PDF) [file pbio.3000298.s008.pdf]

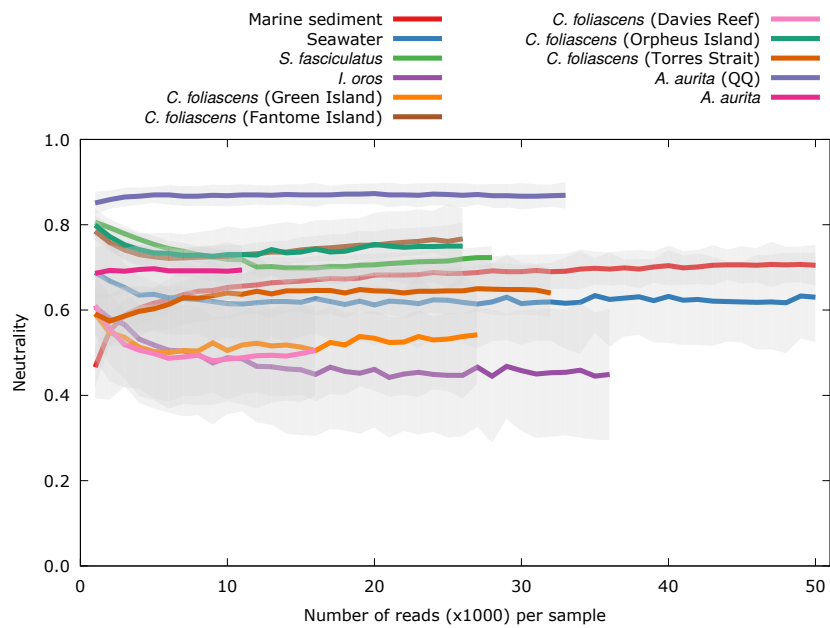

Supplement: S6 Fig — Generally, consistency with the neutral model initially decreased with increasing read depth, until it leveled off as read depth increased further. For communities that showed a high consistency with the neutral model, varying read depths did not affect the results much, ranging from almost no effect at all for A. aurita to a slight drop in neutrality for the seawater samples, from R2 = 0.7 at 1,000 reads/sample to R2 = 0.6 at 50,000 reads/sample. Only for the communities associated with the sponge I. oros and two of the C. foliascens populations did read depth show a more pronounced effect; in both cases, neutrality dropped from R2≈ 0.6 at 1,000 reads/sample to R2≈ 0.45 when read depth was exceeding 10,000 reads/sample. Interestingly, an opposite trend was observed for the sediment samples, in which neutrality increased from R2≈ 0.5 at 1,000 reads/sample to R2≈ 0.7 at 10,000 reads/sample. The minimal to moderate changes in neutrality with increasing read depth for some datasets potentially reflect the influence of rare, non-neutral taxa, which are only detected with higher read depths. Shaded areas indicate 95% bootstrap confidence intervals. The data used to generate this figure are available in S1 Data. (PDF) [file pbio.3000298.s009.pdf]

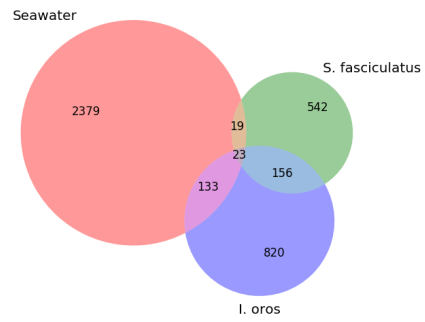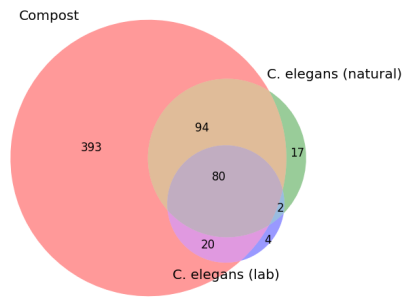

Supplement: S7 Fig — Left: for two sponge species, there is only a very small overlap between the sponge microbiota and the taxa found in seawater. Right: for C. elegans, only a subset of the environmentally available microbes is found in the worms. The OTU abundance data used to generate this figure are available in S2–S10 Data. OTU, operational taxonomic unit. (PDF) [file pbio.3000298.s010.pdf]
